# Genetic Mutation Analysis of High and Low IgY Chickens by Capture Sequencing

**DOI:** 10.3390/ani9050272

**Published:** 2019-05-23

**Authors:** Qiao Wang, Fei Wang, Lu Liu, Qinghe Li, Ranran Liu, Maiqing Zheng, Huanxian Cui, Jie Wen, Guiping Zhao

**Affiliations:** 1 Institute of Animal Sciences, Chinese Academy of Agricultural Sciences, Beijing 100193, China; 17746516692@163.com (Q.W.); feiwang2840@163.com (F.W.); lulu610@zju.edu.cn (L.L.); qli2014@126.com (Q.L.); liuranran@caas.cn (R.L.); zhengmaiqing@caas.cn (M.Z.); cuihuanxian@caas.cn (H.C.); wenjie@caas.cn (J.W.); 2 State Key Laboratory of Animal Nutrition, Beijing 100193, China; 3 College of Animal Science and Technology, Yangzhou University, Yangzhou 225009, China; 4 School of Life Science and Engineering, Foshan University, Foshan 528225, China

**Keywords:** SNP, Indel, White Leghorn, Beijing-You, IgY level

## Abstract

**Simple Summary:**

Immunoglobulin Y (IgY) is the major antibody produced by hens and it endows their offspring with effective humoral immunity against the pathogens. In previous research, we identified 13 genomic regions that were significantly associated with the serum IgY level or antibody responses to sheep red-blood-cells, but the specific mutations in these regions have not been reported. Therefore, we screened for variations in these regions in White Leghorn and Beijing-You chickens with high and low IgY. Our study identified 35,154 mutations and 829 Indels which were associated with IgY levels in both lines. Many non-synonymous mutations were located in crucial genes related to the host immune function, indicating the possible involvement of these genes in controlling IgY levels.

**Abstract:**

Based on the results of our previous genome-wide association study (GWAS), a comprehensive analysis on single nucleotide polymorphisms (SNPs) was performed on White Leghorn and Beijing-You chickens with high and low IgY levels in defined genomic regions using the capture-sequencing approach. High and low IgY chickens showed substantial genetic variations. In total, more than 30,000 SNPs were found in all four chicken groups, among which 1045 were non-synonymous mutations resulting in amino acids alterations. In total, 23,309 Indels were identified. Among the 1169 Indels that were found only in Beijing-You chickens, 702 were shared between high and low IgY chickens compared with the reference genome. There were 1016 Indels specific to the White Leghorn chickens, among which 188 were high IgY-specific, 134 were low IgY-specific and 694 were shared between the high and low IgY chicken lines. Many genetic mutations were located in the regulatory regions of important immunomodulatory genes, including *TAP1*, *TAP2* and *BF1*. Our findings provide an in-depth understanding of genetic mutations in chicken microchromosomes.

## 1. Introduction

The chicken karyotype consists of 39 chromosome pairs, including one sex chromosome pair. With the development of sequencing technology, the chicken W chromosome was sequenced recently [1], but some genomic regions remain poorly annotated due to their complex sequence composition. These include microchromosome 16 (GGA16) and 19 (GGA19). Microchromosomes are denser in genetic material compared with macrochromosomes. The size of GGA16 is estimated at 9–11 Mb, but only 0.5 Mb have been assembled with known sequences [2,3]. GGA16 is of great interest because many genes on this chromosome are closely related to infectious diseases responses. The nucleolus organizing region (NOR), which is a cluster of genes that encode ribosomal RNAs 28S, 18S and 5.8S, is located on GGA16. The NOR copy number and expression level affect cell growth, cell differentiation and protein synthesis capacity [4,5]. The chicken major histocompatibility complex (MHC) has also been mapped to GGA16 [6]. Proteins encoded by the MHC are widely involved in viral and bacterial disease resistance and immune response traits of chickens [7]. Class I and II MHCs include the *BF* and *BL* genes which determine resistance and susceptibility to various diseases [8].The class IV MHC encodes erythrocyte antigens, which are used for MHC haplotype classification. CD1 genes constitute a family of conserved cell-surface proteins with crucial roles in the immune system related to antigen presentation to T cells encoded by MHC [5,9].

There are three types of immunoglobulins, including IgM, IgA and IgY, which have been identified in chickens. IgY is the major antibody in chickens [10]. IgY accumulates in egg yolk and provides the offspring with powerful immunity against avian pathogens [10]. A genome-wide association study (GWAS) was performed for IgY levels and antibody responses to sheep red-blood-cells (SRBC) using the chicken 60k high-density single nucleotide polymorphism (SNP) array [11]. There were five SNPs found to be significantly associated with the serum IgY levels. These SNPs were found on chicken chromosome 16: 5,661,311 to 4,412,041 and chromosome 11: 4,412,041 to 5,661,311 [11]. There were five SNPs in chromosome 19 (spanning 8,974,480 to 9,730,805) and three SNPs in chromosome 11 (spanning 13,531,246 to 14,463,528) which were shown to be associated with antibody responses to sheep red-blood-cells (SRBC). 

In the present study, SNPs were screened in the above-mentioned genomic regions between high and low IgY chickens in the White Leghorn and Beijing-You lines. The capture chip was designed based on all known sequences of chromosome 16 and the genomic regions covering SNPs that have been associated with IgY levels and antibody responses to SRBC in other chromosomes. The captured DNA was then sequenced using high-throughput sequencing technology. Thousands of genetic variations were identified between the high and low IgY chickens, indicating substantial genetic differences between the lines.

## 2. Materials and Methods

### 2.1. Animals and Sample Collection

The animal experiments were carried out in accordance with the Guidelines for Experimental Animals established by the Ministry of Science and Technology (Beijing, China) and the study was approved by the Animal Management Committee of the Institute of Animal Sciences, Chinese Academy of Agricultural Sciences (IAS-CAAS, Beijing, China). Ethical approval regarding animal survival was given by the animal ethics committee of IAS-CAAS (approval number: IASCAAS-AE20140615).

Chickens used in the present study were of the White Leghorn and Beijing-You lines. Beijing-You chickens, who were not subjected to artificial selection, were chosen for this study according to their IgY content. White Leghorn chickens were the result of 7 generations of artificial selection based on anti-SRBC titers. The IgY levels of 527 White Leghorn and 726 Beijing-You chickens were measured by enzyme-linked immune sorbent assay (ELISA). Chickens were divided into high and low IgY groups based on the IgY levels. There was a 5.1-fold difference in the IgY levels between high and low IgY White Leghorn chickens, and a 6.2-fold difference between high-and low-IgY Beijing-You chickens (Table 1).

Forty animals of both varieties with the highest and lowest IgY levels divided into four groups. Each group was randomly and equally divided into four repeats, each containing 10 individuals. Blood was collected from each animal and genomic DNA was isolated using phenol–chloroform extraction. DNA from the 10 individuals of each repeat was pooled together and sent for high-throughput sequencing by HiSeq2500 for SNPs and Indels. 

Genomic DNA was obtained from 34 and 37 animals from lines that were selected for susceptibility and resistance to Marek’s disease respectively, from researchers at Aarhus University (Aarhus, Denmark). This DNA was utilized for sequence analysis of BF1.

### 2.2. ELISA

Serum IgY levels of chickens from both lines were determined using an IgY Chicken ELISA Kit (Abcam) according to the manufacturer’s instructions. 

### 2.3. Capture Sequencing, Sequence Alignment and Data Analysis

The sequence capture chip was designed by Roche Nimblegen to cover a 3.268 Mb region accounting for 97.73% of the 3.371 Mb region associated with the specific phenotypes of interest. Sequences generated by high-throughput sequencing were aligned to the chicken reference genome (galGal4) by SOAP aligner. High confidence SNPs and Indels were identified by SAM tools with the criteria of coverage >4 and FDR < 0.01. Sequences generated by high-throughput sequencing were aligned to the chicken reference genome (galGal4) to identify SNPs and Indels.

### 2.4. Validation of SNPs and Indels

A total of 24 SNPs covering the second-generation sequencing region were randomly selected for validation. A mutation common to both Beijing-You and White Leghorns, plus a unique Indel, with high-IgY were validated. Validation populations were selected from the high-throughput sequence samples. In particular, DNA was pooled from 10 individuals in each group to verify SNPs. Regarding the Indel validation, all sequencing samples were used. Primers were designed around the SNPs and Indels, and PCR amplifications of multiple samples were carried out using High Fidelity PCR Super Mix (Transgen, Biotech). PCR products were sequenced using an ABI PRISM^®^ 3100 Genetic Analyzer. 

## 3. Results

### 3.1. Overall Characteristics of SNPs in High and Low IgY Beijing-You and White Leghorn Chickens 

**Figure 1:**
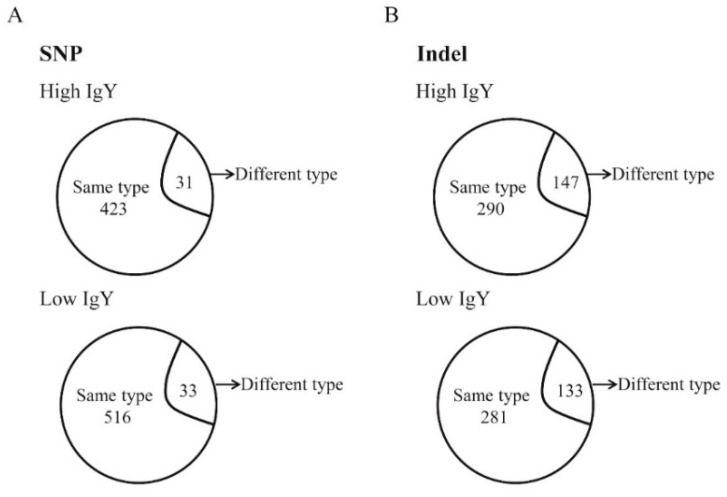
Summary of genetic mutations between high and low IgY White Leghorn and Beijing-You chickens. (**A**) Summary of single nucleotide polymorphisms (SNPs). (**B**) Summary of Indels.

A capture DNA array was designed to cover genomic regions in chromosome 16 and those surrounding significant SNPs associated with IgY levels and antibody responses to SRBC (Table 2). In total, 2.55 Mb of genomic regions were included in our capture chip. There were four duplicates of each chicken line sequenced by Hiseq-2500, and 3.75–7.29 Gb of data was generated for each sample pool. Among all four chicken lines, 35,154 SNPs were identified within the evaluated genomic regions (Table 3). Among these SNPs, 1045 were non-synonymous mutations, resulting in amino acids mutations (Table 3). Regarding the 35,154 SNPs, 1515 were found only in Beijing-You chickens and 727 were shared between high and low IgY chickens compared with the reference genome (Figure 1A and Table S1 in the Supplementary Materials). Further, 3051 SNPs were identified only in White Leghorn chickens, and 2110 of these were shared between high- and low-IgY chickens (Figure 1A and Table S2).

### 3.2. Characteristics of the Indels in High and Low IgY Beijing-You and White Leghorn Chickens

In total, 23,309 Indels were identified (Table 4), and 1169 Indels were found only in Beijing-You chickens. Among these, 702 were shared between high- and low-IgY chickens compared with the reference genome (Figure 1B and Table S3). In Beijing-You chickens, high-IgY specific Indels accounted for 23.5% of the total Indels, and low -IgY specific Indels accounted for 16.4%. There were 1016 Indels identified only in White Leghorn chickens, among which 188 were high-IgY-specific, 134 were low-IgY-specific and 694 were shared between the high -and low-IgY chicken lines (Figure 1B and Table S4).

### 3.3. Validations of SNPs and Indels

Of the SNPs identified by high-throughput sequencing, 87.5% were confirmed (Table 5), indicating the high accuracy of the SNP identifications. A 9 bp deletion with the sequence of CCACTGCCA was found in the intron of *BF1.* The Indels in the *BF1* gene were confirmed in an expanded group of 160 chickens (Table 5).

Further validation of a *BF1* gene deletion was carried out in a chicken population from the Aarhus University selected for Marek’s disease resistance [12]. Genomic DNA of 34 and 37 individuals from the susceptible and the resistant chicken lines respectively, were utilized for sequence analysis of *BF1*, respectively. No deletions were found in either the susceptible or resistant chickens, but a 3 bp mutation was found in both lines. The genotypes of all the chickens from the susceptible chicken line were wild type, but the sequence composition of the 9 bp deletion in the resistant chicken line changed to CTACAGCCC (Table 6).

### 3.4. Comparative Analysis of the Specific SNPs and Indels in the High-IgY White Leghorn and Beijing-You Chicken Lines or Low IgY Counterparts

**Figure 2:**
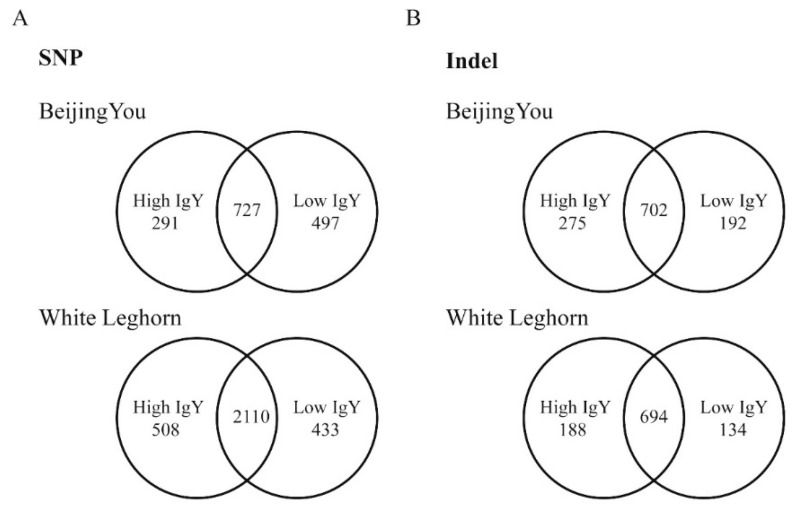
Comparison of genetic mutations between White Leghorn and Beijing-You chickens. Same type: The mutation between these two breeds is the same; different type: The mutation is different. (**A**) SNPs. (**B**) Indels.

Analyses of high-and low-IgY specific SNPs and Indels were performed by comparing sequence differences of high-IgY White Leghorn and Beijing-You chickens with their low-IgY counterparts. It was identified that 423 SNPs related to high-IgY level in both White Leghorn and Beijing-You chickens (Figure 2A and Table S5), among which 31 SNPs shared the same positions but had different mutations in the two high-IgY lines. Regarding low-IgY levels, 516 SNPs were found to be low-IgY-specific (Figure 2A and Table S6) and 33 of these were in the same positions but showed different mutations between the White Leghorn and Beijing-You chicken lines.

A total of 290 Indels were found in both the high-IgY White Leghorn and Beijing-You chicken lines (Figure 2B and Table S7). Two hundred and eighty-one low-IgY specific Indels in the same genomic regions, while 133 Indels were in different positions compared with the high IgY group (Figure 2B and Table S8).

### 3.5. IgY-Related Non-Synonymous SNPs in Coding DNA Sequences and Proximal Promoter Regions

In this study, particular focus was placed on the genetic mutations in coding DNA sequences (CDS) and proximal promoter regions of genes. Therefore, mutations can generate either missense or frame shift mutations in proteins or alter the transcriptional activity of genes. Most chicken gene promoters have not been mapped in detail, and therefore we defined the proximal promoter from the transcription start site (TSS) to 2000bp upstream of the TSS. There were 20 and 19 non-synonymous SNPs identified in CDS regions and 20 and 19 SNPs in proximal promoter regions found in high IgY White Leghorn and Beijing-You chickens (Table 7) respectively. Regarding the above SNPs, 18 in CDS regions and eight in proximal promoters were shared in White Leghorn and Beijing-You chickens (Table 7). There were 21 and 15 non-synonymous SNPs found in CDS regions of low IgY White Leghorn and Beijing-You chickens (Table 6), while nine and 11 SNPs in proximal promoters were identified in low-IgY White Leghorn and Beijing-You chickens respectively (Table 7). Among low-IgY White Leghorn and Beijing-You chickens, 14 and 11 shared SNPs were identified in CDS regions and proximal promoters respectively.

An analysis of genes that harbored the important common SNPs was undertaken in the promoter regions and CDS regions that were shared by both chicken breeds. In high IgY chicken lines, common SNPs in promoters mapped to three genes, including transporter 2 (*TAP2*), AKT interacting protein (*AKTIP*) and TAP binding protein (*TAPBP*) (Table 8). Both TAP2 and TAPBP are closely related to MHC class I molecules and are known to be involved in immune responses in vivo. Common non-synonymous SNPs of high IgY chicken lines in CDS regions are mapped to *BLEC2*(NK receptor-like), MHC BF1 class I(*BF1*), TAP2, tripartite motif containing 41(*TRIM41*), major histocompatibility complex class I antigen BF2 (*BF2*) and transporter 1 (*TAP1*) (Table 8). *TAP2* contained SNPs in both the promoter and CNS regions, indicating a strong selection pressure on this immune-related gene when selecting for high IgY levels.

There were five genes that had common SNPs in their proximal promoter regions in the low-IgY chicken lines, including TAP1, BF2, tumor necrosis factor alpha-induced protein 1 (*TNFAIP1*), major histocompatibility complex class II beta chain (*BMA2*) and *AKTIP.* In the CDS regions, common SNPs were found in *TAP1*, *BF2*, *BF1* and tripartite motif containing 7 (*TRIM7.1*). *TAP1* and *BF2* showed SNPs in both proximal promoter and CDS regions in the low-IgY chicken lines. 

### 3.6. Genomic Distribution of High and Low IgY-Specific Indels 

Compared with SNPs, fewer common Indels were found in the White Leghorn and Beijing-You chicken lines. Only a 9 bp deletion was shared between the high-IgY chicken lines and no shared Indels were found between low-IgY chicken lines (Table 9). This deletion was located in an overlapping *TAP1* 3′UTR and *BF1* genomic sequence and was further validated by Sanger sequencing.

## 4. Discussion

Based on the results of a previous genome-wide association study (GWAS),a capture-sequencing approach was performed to identify variants in defined genomic regions among chickens with high- and low-IgY levels in the White Leghorn and Beijing-You line. In total, 35,154 SNPs and 23,309 Indels were identified. Among these mutations, of particular interest were the genetic mutations in proximal promoters and CDS regions, as such mutations lead to changes in promoter activity or amino acid sequences. Transcription factors can bind to promoter regions and initiate gene transcription [13]. Genetic mutations in these regions can block the binding of transcription factors and thus suppress gene transcriptional activity. Amino acid changes in critical positions may even cause changes in protein structure. There were three and five genes shown to contain SNPs in proximal promoter regions in the high- and low-IgY chickens, among which the *TAP2* gene was identified in both groups. There were five genes, including *TAP2*, *BLEC2*, *BF1*, *BF2*, *TAP1* and *TRIM41*, that have common non-synonymous SNPs in CDS regions in the high IgY group in both White Leghorn and Beijing-You chickens. *TAP1*, *BF1*, *BF2* and *TR*IM7.1 contained non-synonymous SNPs in their CDS regions in the low-IgY groups of both lines. Both the high- and low-IgG groups contained SNPs in the CDS regions of *TAP1, BF1* and *BF2*.

Many of the genes with mutations in the proximal promoter and CDS regions play crucial roles in host immune function. TAP1 forms a heterodimeric complex with TAP2 that has transporter activity. The TAP complex is involved in transporting antigens to the endoplasmic reticulum to associate with MHC class I and promote immune responses against infection [14]. ICP47 encoded by the herpes simplex virus (HSV) is known to inhibit peptide binding to TAP and impairs peptide loading into MHC class I molecules [14,15]. In the present study, SNPs were found in *TAP1* in the CDS regions of high-IgY chickens and in both the CDS regions and proximal promoter regions of low-IgY chickens. SNPs were also found in *TAP2* in CDS regions and proximal promoter, but only in the high IgY chickens. In addition to the SNPs, a 9 bp deletion was identified in the region overlapping the TAP 3′UTR, and the genomic DNA region of *BF1*, indicating the potential impact of *TAP1* and *TAP2* mutations on chicken immune molecules.

The chicken MHC B region encodes class I and II molecules. Different haplotypes determine the resistance or susceptibility to infectious diseases [8]. BF1 and BF2 are encoded by the chicken MHC class I genes. As the dominant MHC class I gene, *BF2* is transcribed more abundantly than *BF1* [16]. Both BF1 and BF2 are deeply involved in antigen processing and immune response. In the present study, SNPs located in both the proximal promoter regions and CDS regions of *BF2* were identified in low-IgY chickens as well as in the CDS regions of *BF2* in high-IgY chickens. In addition to the SNPs, a nine base pairs deletion was identified in the intron of *BF1*. As *BF1* is located in the chicken MHC which has strong effects on host resistance to infectious pathogens, this deletion was examined in well-known chicken selection lines for Marek’s disease resistance from Aarhus University [12]. No deletions were found in either the susceptible or the resistant lines, but SNPs in three bases were identified in the resistant chicken line at the same locus as the 9 bp deletion. These results strongly implicated BF1′s role in resistance to Marek’s disease. 

The two tripartite (TRIM) motif containing proteins, TRIM41 and TRIM7.1, have been shown to contain SNPs in the CDS. The TRIM protein family includes more than 100 members with a conserved TRIM motif and E3 ubiquitin ligase activity [17,18]. Most TRIM proteins are closely associated with the degradation of exogenous and endogenous proteins through the ubiquitin-proteasome pathway. TRIM41 blocks the transcription and replication of hepatitis B virus (HBV) by inhibiting the enhancer II activity of HBV in human hepatoma cells [19]. Previous studies have shown that TRIM7 can promote lung cancer development and a knockdown of TRIM7 reduced tumor growth [20]. TRIM7 and TRIM41 both belong to the TRIM-B30.2 protein family and are located in a region surrounding the chicken BF/BL region of the B complex on chromosome 16 [21]. Non-synonymous SNPs were found in the CDS regions of *TRIM7* and *TRIM41* in low IgY chickens and high IgY chickens respectively, indicating the potential involvement of these genes in the selection for IgY level. 

## 5. Conclusions

In the present study, SNPs and Indels were identified in genomic regions associated with IgY level in chickens. A large number of genetic mutations were identified in the studied genomic regions, revealing abundant genetic diversity between high and low IgY chickens. The chickens used in this study showed substantial differences in IgY levels. In total, it was found that 35,154 and 829 Indels respectively, in the high and low lines. Among them, 18 in CDS regions and 8 in proximal promoters were shared between White Leghorn and Beijing-You chickens with high IgY level. Among low-IgY White Leghorn and Beijing-You chickens, 14 and 11 shared common SNPs in CDS regions and proximal promoters respectively. In addition to the above SNPs common between the two chicken breeds, other identified SNPs and Indels were specific to White Leghorn or Beijing-You chickens, indicating the fundamental role of genetic mutations in modulating antibody levels. This study contributes valuable insights into the understanding of immune gene enriched microchromosome of chickens.

## Figures and Tables

**Table 1:** Averaged immunoglobulin Y (IgY) levels of selected chicken lines.

Chicken Line	Averaged IgY Level (ng/uL)	High IgY Group (ng/uL)	Low IgY Group (ng/uL)	Fold Changes
**White leghorn**	(n = 527)	(n = 40)	(n = 40)	5.1
	679.9 ± 768.17	1501.70 ± 1678.05	293.91 ± 58.29	
**Beijing-You**	(n = 726)	(n = 40)	(n = 40)	6.2
	628.2 ± 923.987	1888.84 ± 1189.55	302.55 ± 24.91	

**Table 2:** Genomic regions covered by sequence capture chip.

Chromosome	Genomic Regions	Related Phenotype	Length (Mb)
11	4,412,041–5,661,311	IgY level	1.25
	13,531,246–14,463,528	antibody responses to SRBC	0.93
16	1–535,270	IgY level	0.54
19	8,974,480–9,730,805	antibody responses to SRBC	0.76

**Table 3:** SNP distribution in different genomic components.

	Homozyg-Ous	Heterozy-Gous	Non-Synonymous	Synonymous	Gene	mRNA
**Number**	28,862	6292	1045	2190	18,332	22,327
**Percent**	82.1%	17.9%	2.9%	6.2%	52.1%	6.5%

**Table 4:** Indels distribution in different genomic components.

	Insertion	Deletion	CDS	UTR	mRNA
**Number**	11,211	12,098	1051	796	11,949
**Percent**	48.1%	51.9%	4.5%	3.4%	51.3%

**Table 5:** Validations of SNPs and Indels by Sanger sequencing.

Position	Mutation Type	Consistence with High-Throughput Sequencing
Chr16: 213,135	C/G	Yes
Chr16: 213,142	A/G	Yes
Chr16: 213,221	T/G	Yes
Chr16: 213,160	T/G	No
Chr16: 61,196	C/G	Yes
Chr16: 61,211	A/C	Yes
Chr11: 4,464,202	G/A	Yes
Chr11: 4,478,491	C/A	Yes
Chr11: 4,478,592	T/C	Yes
Chr11: 4,478,634	T/C	Yes
Chr11: 4,480,688	G/T	Yes
Chr11: 4,480,721	C/A	Yes
Chr11: 4,479,104	C/T	Yes
Chr11: 4,479,176	G/A	Yes
Chr11: 4,479,218	A/G	Yes
Chr11: 4,479,254	C/T	Yes
Chr11: 4,480,840	C/T	Yes
Chr11: 4,480,934	T/A	Yes
Chr11: 4,480,961	G/A	Yes
Chr11: 4,480,971	G/A	Yes
Chr11: 4,481,057	G/A	Yes
Chr11: 4,481,115	A/G	Yes
Chr11: 4,478,592	T/C	No
Chr11: 4,478,634	T/C	No
Chr16:74,016–74,024	CCACTGCCA, deletion	Yes

**Table 6:** Frequencies of 9 bp deletions in BF1 gene in lines of chickens selected for resistance to Marek’s disease.

Chicken Line	CCACTGCCA	CTACAGCCC
The susceptible lines	100%	0
The resistant lines	0	100%

**Table 7:** Number of non-synonymous SNPs in the coding DNA sequences and proximal promoter regions.

Position	Coding DNA Sequence	Proximal Promoter
White Leghorn high IgYgroup	20	11
Beijing-You high IgY line	19	10
Common SNPs in high IgY White Leghorn and Beijing-You chickens	18	8
White Leghorn low IgYgroup	21	9
Beijing-You low IgY line	15	11
Common SNPs in low IgY White Leghorn and Beijing-You chickens	14	11

**Table 8:** Genes with common SNPs to the high IgY groups or low IgY groups in proximal promoter or CDS.

Genes with Common SNPs of High IgY Groups		Genes with Common SNPs of Low IgY Groups	
Proximal Promoter	CDS	Proximal Promoter	CDS
TAP2	TAP2	TAP1	TAP1
AKTIP	BLEC2	BF2	BF2
TAPBP	BF1	TNFAIP1	BF1
	BF2	DMB2	TRIM7
	TAP1	AKTIP	
	TRIM41		

**Table 9:** Indel common to the high IgY groups or low IgY groups.

Indel Type	Sequence	Gene	Position
Deletion	CCACTGCCA	BF1	Intron
		TAP1	3′UTR

## Supplementary Materials

The following are available online at https://www.mdpi.com/2076-2615/9/5/272/s1. Table S1: the unique SNPs in Beijing-You chickens, Table S2: the unique SNPs in White Leghorn chickens, Table S3: the unique Indels in Beijing-You chickens, Table S4: the unique Indels in White Leghorn chickens, Table S5: SNPs related to high-IgY level in both varieties, Table S6: SNPs related to low-IgY level in both varieties, Table S7: Indels related to high-IgY level in both varieties, Table S8: Indels related to low-IgY level in both varieties.
